# Risk Factors for Cutaneous Immune‐Related Adverse Events in the Japanese Population: A Retrospective Single‐Center Cohort Study

**DOI:** 10.1111/1346-8138.70370

**Published:** 2026-07-02

**Authors:** Tomoya Watanabe, Kohei Yamakawa, Miyu Kobayashi, Yusuke Saigusa, Yukie Yamaguchi

**Affiliations:** ^1^ Department of Environmental Immuno‐Dermatology Yokohama City University Graduate School of Medicine Yokohama Japan; ^2^ Department of Biostatistics Yokohama City University School of Medicine Yokohama Japan

**Keywords:** cohort study, cutaneous immune‐related adverse events, immune checkpoint inhibitors, Japanese population, risk factors

## Abstract

Immune checkpoint inhibitor (ICI)‐driven alterations in the immune system can induce immune‐related adverse events (irAEs). Cutaneous irAEs are notable for being the most common and for their early onset; however, the risk factors underlying their development and severity remain unclear. In this study, we retrospectively analyzed 1224 Japanese patients treated with at least one ICI at Yokohama City University Hospital between 2014 and 2024 and identified the risk factors for cutaneous irAEs using multivariable logistic regression analysis. Overall, 145 cutaneous irAEs were identified in 134 patients (10.9%), of which 18 (1.5%) developed grade 4 cutaneous irAEs. Statistical analysis revealed that anti‐programmed cell death protein 1 (anti‐PD‐1) and anti‐cytotoxic T‐lymphocyte associated protein‐4 (anti‐CTLA‐4) combination therapy was significantly associated with the risk of developing cutaneous irAEs compared with anti‐PD‐1 (odds ratio [OR] = 3.21; 95% confidence interval [CI], 1.85–5.54; *p* < 0.001) or anti‐PD ligand 1 (OR = 4.53; 95% CI, 2.06–10.34; *p* < 0.001) monotherapy. A higher baseline eosinophil count (OR = 1.27; 95% CI, 1.06–1.55; *p* = 0.013) and melanoma (OR = 2.11; 95% CI, 1.17–3.72; *p* = 0.011) were associated with an increased risk of cutaneous irAEs. Furthermore, female sex (OR = 4.13; 95% CI, 1.36–13.11; *p* = 0.012) and high baseline monocyte count (OR = 6.68; 95% CI, 1.87–27.61; *p* = 0.003) were risk factors for grade 4 cutaneous irAEs. These results indicated that anti‐PD‐1 and anti‐CTLA‐4 combination therapy, high eosinophil counts, and melanoma were associated with a higher risk of developing cutaneous irAEs, whereas female sex and high monocyte counts were risk factors for severe cutaneous irAEs.

## 1 Introduction

Immune checkpoint inhibitors (ICIs), which block key mediators of tumor‐mediated immune evasion, have been increasingly used to treat numerous malignancies since their initial approval for treating patients with unresectable melanoma in 2014. ICIs targeting immune checkpoint molecules, such as programmed cell death protein 1 (PD‐1), PD ligand 1 (PD‐L1), and cytotoxic T‐lymphocyte‐associated protein‐4 (CTLA‐4), which all modulate T‐cell activation, have shown remarkable efficacy against many malignancies [1]. ICI‐induced alterations in the immune system can cause various immune‐related adverse events (irAEs), resulting in significant morbidity and treatment discontinuation [2]. IrAEs affect multiple organ systems such as the thyroid gland, adrenal glands, skin, gut, muscles, liver, and lungs [3, 4, 5]. Among these, cutaneous irAEs are the most common and often the first to develop [6]. Therefore, identifying the risk factors for cutaneous irAEs may contribute to treatment adherence and improve patient survival. A previous report comparing the incidence of irAEs in three tumors (melanoma, non‐small cell lung cancer [NSCLC], and renal cancer) treated with anti‐PD‐1 antibodies reported a higher frequency of cutaneous irAEs in patients with melanoma [7]. Furthermore, correlations between cutaneous irAEs and melanoma and respiratory irAEs and NSCLC have been identified, indicating that some irAEs may be caused by a specific response to primary tumors [8]. However, the risk factors for the development and severity of cutaneous irAEs remain unclear.

In this study, we aimed to retrospectively investigate the risk factors for the development and severity of cutaneous irAEs in Japanese patients with malignancies treated with ICIs.

## 2 Methods

### 2.1 Patients and Study Design

**FIGURE 1:**
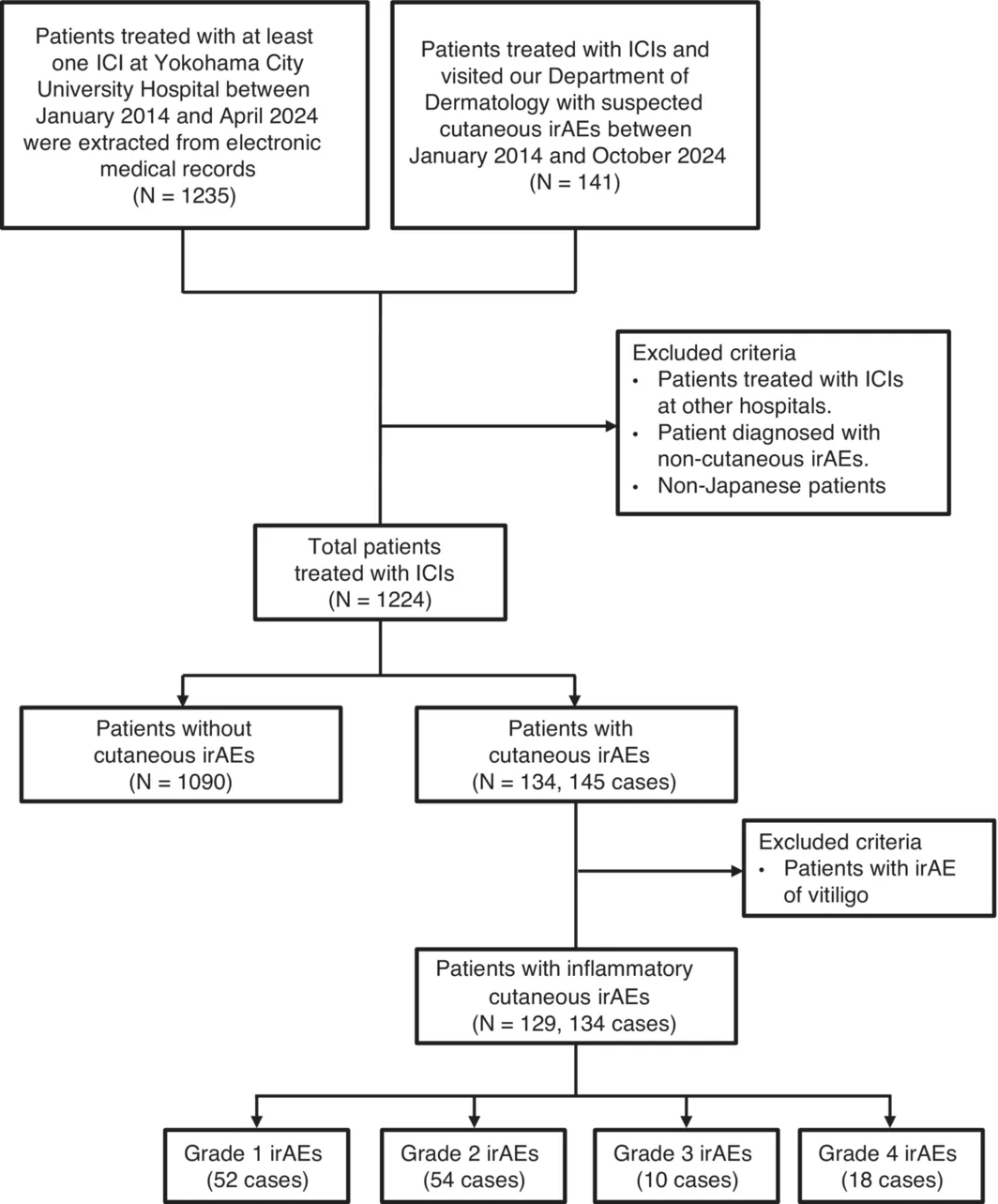
Flowchart of study design. A flowchart of patients with and without cutaneous irAEs is shown. Patients with cutaneous irAEs are further divided into those with grades 1–4. irAEs, immune‐related adverse events.

Data of patients treated with at least one ICI, including nivolumab (PD‐1 inhibitor), pembrolizumab (PD‐1 inhibitor), atezolizumab (PD‐L1 inhibitor), durvalumab (PD‐L1 inhibitor), avelumab (PD‐L1 inhibitor), or ipilimumab (CTLA‐4 inhibitor) at Yokohama City University Hospital between January 2014 and April 2024 were extracted from electronic medical records. Patients who visited the Department of Dermatology with suspected cutaneous irAEs were included. Furthermore, patients who received ICIs at other hospitals, those diagnosed with noncutaneous irAEs by dermatologists, and 11 non‐Japanese patients (to exclude racial influence) were excluded from this study. In the analysis of cutaneous irAE severity, patients with vitiligo were excluded to focus specifically on inflammatory cutaneous irAEs. The data collected were integrated and analyzed (Figure 1). This study was approved by the Institutional Review Board of Yokohama City University (approval no.: F241100033).

### 2.2 Clinical and Biological Data

Clinical and biological data were collected until October 2024 from patients who received ICIs in April 2024 to observe the onset of irAEs. The collected data included age, height, weight, and body mass index (BMI) at the time of first ICI administration and at the onset of cutaneous irAEs, sex, race, type of malignancy, type of ICI, prior chemotherapy before ICI initiation, concurrent chemotherapy, duration from administration to the onset of cutaneous irAEs, type of cutaneous irAEs, blood sampling data (the number of white blood cells [WBC], neutrophils, lymphocytes, monocytes, eosinophils, and platelets, C‐reactive protein, and albumin) at the initiation of ICIs (baseline) and the onset of irAEs, history of allergic diseases (atopic dermatitis, pollen allergies, asthma, rhinitis, conjunctivitis, drug allergies, food allergies), computed tomography (CT) scan data, treatment details of cutaneous irAEs, duration of treatment, and outcome. BMI was calculated as weight/height^2^ (kg/m^2^). All baseline laboratory data were obtained within 2 weeks prior to ICI initiation. Prior chemotherapy was defined as non‐ICI treatment, including cytotoxic chemotherapy, administered within 3 months before ICI initiation to account for the potential carryover effects of recent chemotherapy on baseline peripheral blood cell counts [9, 10]. Based on World Health Organization guidelines, patients were classified into underweight (BMI = < 18.5 kg/m^2^), normal‐weight (BMI = 18.5–24.9 kg/m^2^), overweight (BMI = 25.0–29.9 kg/m^2^), or obese (BMI ≥ 30.0 kg/m^2^) [11]. The types of ICIs were further divided into three groups: PD‐1 inhibitors (nivolumab and pembrolizumab), PD‐1 inhibitors with CTLA‐4 inhibitors (nivolumab and ipilimumab), and PD‐L1 inhibitors (atezolizumab, durvalumab, and avelumab). The type of irAE was defined as those affecting the (1) endocrine system (primary hypothyroidism, thyrotoxicosis, primary adrenal insufficiency, hypophysitis, and diabetes), (2) skin, (3) lung (pneumonitis), (4) hepato‐biliary‐pancreatic (hepatitis, cholangitis, and pancreatitis), (5) intestine (colitis), (6) musculoskeletal system (myasthenia gravis, myasthenic syndrome, and myositis), (7) kidney (nephritis and acute kidney injury), (8) joint (arthritis), (9) heart (myocarditis and pericarditis), (10) nervous system (peripheral neuropathy and mononeuritis multiplex), and (11) eye (uveitis, iritis, and episcleritis). In addition, the types of cutaneous irAEs were classified as follows: (1) maculopapular rash, (2) eczematous conditions, (3) erythema multiforme, (4) lichen planus, (5) psoriasiform eruption, (6) Stevens–Johnson syndrome (SJS)/toxic epidermal necrolysis (TEN), (7) bullous diseases, (8) purpuric eruption, (9) vitiligo, and (10) others (alopecia areata, pruritus, xerosis, follicular dermatitis, purpura, and urticarial eruption). The severity of cutaneous irAEs was evaluated based on the grading system established by the American Society of Clinical Oncology [12] with some modifications. This grading system is based on body surface area, tolerability, morbidity, and duration. Considering treatment options, including continuation of ICIs and initiation of systemic corticosteroids therapy, as well as hospitalization or not, we defined grades 1 and 2 as those requiring topical corticosteroids with or without antihistamines with ICI continuation, grade 3 as those requiring prednisolone (PSL) ≤ 0.5 mg/kg and with ICI continuation, and grade 4 as those requiring PSL > 0.5 mg/kg with conditions requiring hospitalization, including SJS and TEN, and ICI discontinuation.

### 2.3 Image Analysis

To calculate the skeletal muscle index (SMI), CT images of the third lumbar vertebra (L3) were analyzed using SYNAPSE Vincent (FUJI Film, Tokyo, Japan). All CT images were obtained within 2 months of ICIs initiation. Hounsfield unit (HU) thresholds were used as follows: −29 to 150 HU for skeletal muscle and −150 to −30 HU for adipose tissue [13, 14]. The skeletal muscle area (cm^2^) was normalized to the height (m^2^) to calculate the SMI (cm^2^/m^2^). Based on the definition of the Japan Society of Hepatology, sarcopenia was defined as an SMI at the L3 level of < 42 cm^2^/m^2^ in males and 38 cm^2^/m^2^ in females [15].

### 2.4 Statistical Analysis

Normally distributed continuous variables were expressed as mean ± standard deviation. Non‐normal continuous variables are expressed as medians with interquartile ranges. Fisher's exact test and the Mann–Whitney *U* test were used for statistical analysis, as appropriate. We performed multivariable logistic regression analyses to investigate the risk factors for the development and severity of cutaneous irAEs. Sex, age at initiation of ICI therapy, BMI, type of malignancy, type of ICI, chemotherapy prior to ICI administration, and laboratory parameters (eosinophil, lymphocyte, and monocyte counts) were included as potential risk factors. In the analysis of the severity of cutaneous irAEs, Firth's correction was applied to address the separation issues caused by the small number of patients assessed as having grade 4 cutaneous irAEs. Multiple events of cutaneous irAEs in the same patient were treated as independent clinical episodes and analyzed as separate observations. All statistical analyses were conducted using GraphPad Prism version 8.2 software (GraphPad Software, San Diego, CA, USA), JMP Pro 16.1.0 software (SAS Institute Inc., Cary, NC, USA), and R version 4.4.2 (R Foundation for Statistical Computing, Vienna, Austria).

## 3 Results

### 3.1 Baseline Patient Profiles

A total of 1224 patients with malignancies who received at least one ICI were included in this study (Figure 1). The median age at first ICI administration was 70 years (range, 29–92). The cohort included 870 males (71.1%) and 354 females (28.9%). Clinical and biological data are summarized in Table 1. A total of 1292 ICI treatment courses were identified. Sixty‐eight patients (6.0%) were treated with two or three courses of ICIs. The most common ICI administered was nivolumab (544 courses, 42.1%), followed by pembrolizumab (425 courses, 32.9%), atezolizumab (115 courses, 8.9%), nivolumab with ipilimumab (108 courses, 8.4%), durvalumab (88 courses, 6.8%), avelumab (8 courses, 0.6%), and ipilimumab (4 courses, 0.3%). Analysis of the number of each type of irAE showed that endocrine irAEs were the most common (209 patients, 17.1%), and cutaneous irAEs were the second most common. The incidence rate of cutaneous irAEs was 10.9% (134/1224). Eleven patients developed two or three different cutaneous irAEs during the ICI treatment period, resulting in a total of 145 cutaneous irAEs. The clinical subtypes and combinations of cutaneous irAEs are summarized in Table S1. The incidence of grade 4 irAEs, representing the most severe category, was 1.5% (18 cases). Previous reports have shown that cutaneous irAEs are often the first to develop among all irAEs [6]. We evaluated whether a similar pattern occurred in our study and found that cutaneous irAEs were the first to appear in 123 patients (91.8%). Among these, four patients simultaneously developed other irAEs, including three endocrine and one pulmonary irAE. These findings confirm that cutaneous irAEs tend to be the earliest to occur. Regarding malignancy type, the most common diagnosis was renal cancer (171 patients, 14.0%), followed by lung cancer (142 patients, 11.6%), melanoma (124 patients, 10.3%), esophageal cancer (111 patients, 9.1%), gastric cancer (107 patients, 8.7%), oral cancer (102 patients, 8.3%), and pharyngeal cancer (101 patients, 8.3%).

**TABLE 1:** Characteristics of 1224 malignancy patients treated with at least one ICIs.

Variable	
Gender	
Male, *n* (%)	870 (71.1)
Female, *n* (%)	354 (28.9)
Age at initiation of ICI administration (years), median (IQR)	70.0 (61.5–76.0)
Type of irAEs, *n* (%)	
Endocrine system	209 (17.1)
Skin	134 (10.9)
Lung	76 (6.2)
Hepato‐biliary‐Pancreatic	69 (5.6)
Intestine	54 (4.4)
Musculoskeletal system	17 (1.3)
Kidney	17 (1.4)
Joint	15 (1.2)
Heart	6 (0.5)
Nervous system	3 (0.2)
Eye	2 (0.2)
Grade of cutaneous irAEs	
irAE (−), *n* (%)	1090 (89.1)
irAE (+), *n* (%)	134 (10.9)
Grade 1 irAE, *n* (%)	57 (5.1)
Grade 2 irAE, *n* (%)	54 (4.4)
Grade 3 irAE, *n* (%)	10 (0.8)
Grade 4 irAE, *n* (%)	18 (1.5)
Number of cutaneous irAEs complications	
Single cutaneous irAEs, *n*	123
Double cutaneous irAEs, *n*	10
Triple cutaneous irAEs, *n*	1
Number of ICIs courses	
Total	1292
Single course, n	1156
Double course, n	65
Triple course, n	3
Type of ICIs	
Nivolumab, *n* (%)	544 (42.1)
Pembrolizumab, *n* (%)	425 (32.9)
Atezolizumab, *n* (%)	115 (8.9)
Durvalumab, *n* (%)	88 (6.8)
Avelumab, *n* (%)	8 (0.6)
Ipilimumab, *n* (%)	4 (0.3)
Nivolumab+Ipilimumab, *n* (%)	108 (8.4)
Type of malignancies, *n* (%)	
Renal cancer	171 (14.0)
Lung cancer	142 (11.6)
Malignant melanoma	124 (10.3)
Esophageal cancer	111 (9.1)
Gastric cancer	107 (8.7)
Oral cancer	102 (8.3)
Pharyngeal cancer	101 (8.3)
Hepatocellular carcinoma	78 (6.4)
Bladder cancer	52 (4.2)
Bile duct cancer	39 (3.2)
Cervical cancer	35 (2.9)
Paranasal cancer	29 (2.4)
Uterine cancer	14 (1.1)
Colorectal cancer	14 (1.1)
Breast cancer	14 (1.1)
Ureteral cancer	14 (1.1)
Cancer of unknown primary	11 (0.9)
Neuroendocrine carcinoma	11 (0.9)
Gallbladder cancer	8 (0.7)
Laryngeal cancer	7 (0.6)
Prostate cancer	7 (0.6)
Parotid cancer	5 (0.4)
Pleural mesothelioma	5 (0.4)
Submandibular adenocarcinoma	4 (0.3)
Malignant lymphoma	3 (0.2)
Duodenal papillary cancer	3 (0.2)
Salivary gland cancer	3 (0.2)
Squamous cell carcinoma	2 (0.2)
Polymorphous adenocarcinoma	1 (0.1)
Merkel cell carcinoma	1 (0.1)
Lacrimal gland cancer	1 (0.1)
Cancer of external auditory cana	1 (0.1)
Sebaceous carcinoma	1 (0.1)
Small intestine cancer	1 (0.1)
Neuroblastoma	1 (0.1)
Pancreatic cancer	1 (0.1)
Patients' treatment histories, *n* (%)	
Chemotherapy prior to ICIs initiation	466 (38.1)
Combination with chemotherapy at the initiation of ICIs treatment	394 (32.2)

Abbreviations: ICIs, immune checkpoint inhibitors; IQR, interquartile range; irAE, immune‐related adverse events.

### 3.2 Risk Factors for the Development of Cutaneous irAEs

To investigate the characteristics of patients with cutaneous irAEs, we summarized and compared the clinical features of patients with and without cutaneous irAEs (Table 2). The age at initiation of ICI administration in patients with cutaneous irAEs (cutaneous irAEs group) was significantly lower than that in patients without cutaneous irAEs (non‐cutaneous irAEs group). Both groups were male‐dominated, with no significant sex difference. The BMI tended to be higher in the cutaneous irAE group than in the non‐cutaneous irAE group, although the difference was not statistically significant. In the analysis of the type of ICIs, the cutaneous irAEs group had a lower percentage of patients treated with nivolumab (33.1% vs. 43.3%) and atezolizumab (4.5% vs. 9.5%) but a higher percentage of patients treated with nivolumab with ipilimumab (24.8% vs. 6.1%) compared with the non‐cutaneous irAEs group. Furthermore, analysis of malignancies treated with ICIs revealed higher rates of melanoma (20.1% vs. 8.9%) and renal cancer (23.9% vs. 12.8%) in the cutaneous irAEs group. In contrast, a low incidence of cutaneous irAEs has been observed in patients with oral, esophageal, and gastric cancers. Furthermore, in the analysis of the number of complicated irAEs, excluding cutaneous irAEs, the cutaneous irAE group had a significantly higher number of complications than the non‐cutaneous irAE group. Meanwhile, analysis of types of irAE complications, excluding cutaneous irAEs, showed a more frequent occurrence of complicated endocrine irAEs in both groups; however, the second most frequent complications were hepato‐biliary‐pancreatic irAEs in the cutaneous irAEs group (13.4%) and pulmonary irAEs in the non‐cutaneous irAE group (6.0%). Analysis of laboratory data showed that baseline eosinophil and lymphocyte counts were significantly higher in the cutaneous irAE group than in the non‐cutaneous irAE group. The prevalence of a history of allergies was similar in both groups, suggesting that allergic conditions did not contribute to the elevated eosinophil counts in the cutaneous irAE group. We also compared baseline eosinophil counts among the different subtypes of cutaneous irAEs. Although eosinophil counts tended to be higher in patients with SJS/TEN and bullous diseases, no statistically significant differences were observed compared with the other subtypes (Table S2 and Figure S1). Regarding treatment history, the proportion of patients who had received chemotherapy prior to ICI initiation was significantly lower in the cutaneous irAE group than in the non‐cutaneous irAE group (25.4% vs. 39.6%).

**TABLE 2:** Comparison of clinical characteristics between patients with and without cutaneous irAEs.

Variable	Patients with cutaneous irAEs (*N* = 134)	Patients without cutaneous irAEs (*N* = 1090)	*p*
Gender, *n* (%)			
Male	102 (76.1)	768 (70.5)	0.190
Female	32 (23.9)	322 (29.5)	
Age (years), median (IQR)	66.0 (59.3–74.0)	70.0 (62.0–76.0)	0.010
BMI (mean ± SD)	22.6 ± 4.0	21.7 ± 3.8	0.039
History of allergy, *n* (%)	36 (26.9)	269 (24.7)	0.581
Number of complicated irAEs excluding cutaneous irAEs, *n* (%)	63 (47.0)	311 (28.5)	< 0.001
Type of irAE			
Skin	134 (100)	0	
Endocrine system	34 (25.4)	175 (16.1)	
Hepato‐biliary‐Pancreatic	18 (13.4)	51 (4.7)	
Lung	11 (8.2)	65 (6.0)	
Intestine	10 (7.5)	44 (4.0)	
Joint	3 (2.2)	12 (1.1)	
Musculoskeletal system	3 (2.2)	14 (1.3)	
Kidney	2 (1.5)	15 (1.4)	
Heart	1 (0.7)	5 (0.5)	
Number of Complication with irAEs			
Single	70 (52.2)	244 (22.4)	
Double	38 (28.4)	56 (5.1)	
Triple	21 (15.7)	5 (0.4)	
Quadruple	4 (3.0)	2 (0.2)	
Type of ICIs			
Total, n	157	1135	
Nivolumab, *n* (%)	52 (33.1)	492 (43.3)	
Pembrolizumab, *n* (%)	49 (31.2)	376 (33.1)	
Atezolizumab, *n* (%)	7 (4.5)	108 (9.5)	
Durvalumab, *n* (%)	8 (5.1)	80 (7.0)	
Avelumab, *n* (%)	1 (0.6)	7 (0.6)	
Ipilimumab, *n* (%)	1 (0.6)	3 (0.3)	
Nivolumab + Ipilimumab, *n* (%)	39 (24.8)	69 (6.1)	
Type of malignancies, *n* (%)			
Renal cancer	32 (23.9)	139 (12.8)	
Lung cancer	15 (11.2)	127 (11.7)	
Malignant melanoma	27 (20.1)	97 (8.9)	
Esophageal cancer	7 (5.2)	104 (9.5)	
Gastric cancer	7 (5.2)	100 (9.2)	
Oral cancer	4 (3.0)	98 (9.0)	
Pharyngeal cancer	12 (9.0)	89 (8.2)	
Hepatocellular carcinoma	7 (5.2)	71 (6.5)	
Bladder cancer	5 (3.7)	47 (4.3)	
Bile duct cancer	3 (2.2)	36 (3.3)	
Cervical cancer	6 (4.5)	29 (2.7)	
Paranasal cancer	2 (1.5)	27 (2.5)	
Uterine cancer	1 (0.7)	13 (1.2)	
Colorectal cancer	1 (0.7)	13 (1.2)	
Breast cancer	0 (0)	14 (1.3)	
Cancer of unknown primary	0 (0)	11 (1.0)	
Ureteral cancer	0 (0)	14 (1.3)	
Neuroendocrine carcinoma	1 (0.7)	10 (0.9)	
Gallbladder cancer	0 (0)	8 (0.7)	
Laryngeal cancer	0 (0)	7 (0.6)	
Prostate cancer	0 (0)	7 (0.6)	
Parotid cancer	0 (0)	5 (0.5)	
Pleural mesothelioma	1 (0.7)	4 (0.4)	
Malignant lymphoma	2 (1.5)	1 (0.1)	
Submandibular adenocarcinoma	0 (0)	4 (0.4)	
Duodenal papillary cancer	0 (0)	3 (0.3)	
Squamous cell carcinoma	0 (0)	2 (0.2)	
Salivary gland cancer	0 (0)	3 (0.3)	
Polymorphous adenocarcinoma	0 (0)	1 (0.1)	
Merkel cell carcinoma	0 (0)	1 (0.1)	
Lacrimal gland cancer	0 (0)	1 (0.1)	
Cancer of external auditory cana	0 (0)	1 (0.1)	
Sebaceous carcinoma	0 (0)	1 (0.1)	
Small intestine cancer	0 (0)	1 (0.1)	
Neuroblastoma	1 (0.7)	0 (0)	
Pancreatic cancer	0 (0)	1 (0.1)	
Laboratory data (mean ± SD)			
White blood cells (/μL)	6813 ± 3281	6583 ± 3285	0.426
Neutrophils (/μL)	4692 ± 3120	4562 ± 2905	0.974
Lymphocytes (/μL)	1323 ± 555	1218 ± 579	0.008
Monocytes (/μL)	552 ± 251	572 ± 297	0.638
Eosinophils (/μL)	212 ± 234	173 ± 322	< 0.001
Platelet (/μL)	278 000 ± 138 000	269 000 ± 117 000	0.975
CRP (mg/dL)	2.06 ± 4.22	2.08 ± 4.04	0.340
Albumin (g/dL)	3.8 ± 0.7	3.7 ± 0.6	0.026
NLR	4.7 ± 5.5	4.9 ± 7.4	0.122
LMR	2.8 ± 1.5	2.5 ± 2.1	0.018
CAR	0.9 ± 2.4	0.7 ± 1.8	0.272
PLR	272 ± 288	278 ± 236	0.156
ELR	0.17 ± 0.20	0.16 ± 0.24	0.006
EMR	0.41 ± 0.46	0.34 ± 0.73	< 0.001
Patients' treatment histories, *n* (%)			
Chemotherapy prior to ICIs initiation	34 (25.4)	432 (39.6)	0.001
Combination with chemotherapy at the initiation of ICIs treatment	37 (27.6)	357 (32.8)	0.329
Outcome			
Dead	68 (50.7)	659 (60.5)	
Survive	65 (48.5)	406 (37.2)	
Unknown	1 (0.8)	25 (2.3)	

Abbreviations: BMI, body mass index; CAR, CRP‐to‐albumin ratio; ELR, eosinophil‐to‐lymphocyte ratio; EMR, eosinophil‐to‐monocyte ratio; ICIs, immune checkpoint inhibitors; IQR, interquartile range; irAE, immune‐related adverse events; LMR, lymphocyte‐to‐monocyte ratio; NLR, neutrophil‐to‐lymphocyte ratio; PLR, platelet‐to‐lymphocyte ratio; SD, standard deviation.

To identify the risk factors for the development of cutaneous irAEs, we performed a multivariable analysis (Table 3). Statistical analyses revealed that combination therapy with anti‐PD‐1 and anti‐CTLA‐4 was associated with a significantly higher risk of developing cutaneous irAEs than anti‐PD‐1 (odds ratio [OR] = 3.21; 95% confidence interval [CI], 1.85–5.54; *p* < 0.001) or anti‐PD‐L1 (OR = 4.53; 95% CI, 2.06–10.34; *p* < 0.001) monotherapy. In addition, melanoma (OR = 2.11; 95% CI, 1.17–3.72; *p* = 0.011) and a higher baseline peripheral blood eosinophil count (OR = 1.27; 95% CI, 1.06–1.55; *p* = 0.013) were significantly associated with the development of cutaneous irAEs. Type IV hypersensitivity reactions have been proposed as one of several mechanisms underlying certain subtypes of cutaneous irAEs [16]. Accordingly, our results suggest that an elevated baseline eosinophil count in the peripheral blood may be involved in the induction of cutaneous irAEs via allergic mechanisms.

**TABLE 3:** Multivariable logistic regression for the risk of the development of cutaneous irAEs.

Variable	OR	95% CI	*p*
Female	0.80	0.51–1.23	0.322
Age at initiation of ICI administration	0.98	0.97–1.00	0.068
BMI	1.03	0.97–1.08	0.316
Type of malignancies			
Renal cancer (vs others)	1.48	0.84–2.54	0.167
Lung cancer (vs others)	1.30	0.68–2.35	0.408
Malignant melanoma (vs others)	2.11	1.17–3.72	0.011
Type of ICIs			
PD‐1 + CTLA‐4 vs. PD‐1	3.21	1.85–5.54	< 0.001
PD‐1 + CTLA‐4 vs. PD‐L1	4.53	2.06–10.34	< 0.001
PD‐1 vs. PD‐L1^a^	1.41	0.77–2.78	0.293
Laboratory data^b^			
Eosinophils	1.27	1.06–1.55	0.013
Lymphocytes	1.07	0.72–1.63	0.737
Treatment histories			
Chemotherapy prior to ICIs initiation	0.750	0.48–1.16	0.199

Abbreviations: BMI, body mass index; CI, confidence interval; CTLA‐4, Cytotoxic T‐lymphocyte Antigen‐4; ICIs, immune checkpoint inhibitors; irAE, immune‐related adverse events; OR, odds ratio; PD‐1, Programmed cell Death 1; PD‐L1, Programmed cell Death ligand 1.

^a^ PD‐1 vs. PD‐L1 was obtained from the same model.

^b^ Eosinophils and lymphocytes were log(1 + x) transformed.

We further investigated the changes in laboratory data at baseline and at the onset of the cutaneous irAEs (Table 4). Albumin, platelet, and lymphocyte counts were significantly decreased, whereas eosinophil counts were significantly increased at the onset of cutaneous irAEs. The decreases in albumin, platelet, and lymphocyte counts were considered to be associated with malignancy progression [17, 18], whereas eosinophil counts were significantly increased at the onset of cutaneous irAEs. This increase may reflect immune activation associated with the development of cutaneous irAEs; however, it does not necessarily indicate a specific allergic mechanism.

**TABLE 4:** Analysis of changes in blood sampling parameters during the initiation of ICI administration and the onset of cutaneous irAEs in 134 patients with cutaneous irAEs.

Variable (mean ± SD)	At the initiation of ICI administration	At the onset of cutaneous irAE	*p*
White blood cells (/μL)	6777 ± 3237	6443 ± 2362	0.671
Neutrophils (/μL)	4654 ± 3076	4179 ± 2362	0.335
Lymphocytes (/μL)	1328 ± 553	1254 ± 595	0.032
Monocytes (/μL)	548 ± 248	566 ± 274	0.223
Eosinophils (/μL)	212 ± 231	384 ± 470	< 0.001
Platelet (/μL)	278 000 ± 136 000	249 000 ± 112 000	0.005
CRP (mg/dL)	2.01 ± 4.16	2.52 ± 4.40	0.102
Albumin (g/dL)	3.8 ± 0.7	3.6 ± 0.7	0.012
NLR	4.6 ± 5.4	4.9 ± 8.9	0.374
LMR	2.8 ± 1.5	2.6 ± 1.5	0.054
CAR	0.8 ± 2.3	0.9 ± 2.1	0.078
PLR	271 ± 283	289 ± 421	0.557
ELR	0.17 ± 0.19	0.35 ± 0.43	< 0.001
EMR	0.42 ± 0.46	0.73 ± 0.83	< 0.001

Abbreviations: CAR, CRP‐to‐albumin ratio; ELR, eosinophil‐to‐lymphocyte ratio; EMR, eosinophil‐to‐monocyte ratio; ICIs, immune checkpoint inhibitors; irAE, immune‐related adverse events; LMR, lymphocyte‐to‐monocyte ratio; NLR, neutrophil‐to‐lymphocyte ratio; PLR, platelet‐to‐lymphocyte ratio.

### 3.3 Risk Factors for Severe Cutaneous irAEs

Next, we examined the risk factors for severe cutaneous irAEs. To focus on inflammatory cutaneous irAEs, patients with vitiligo were excluded from this analysis. A total of 129 patients with 134 cutaneous irAEs were included and classified according to grades 1–4, as summarized in Table 5. Overall, the average interval between the initiation of ICI and the onset of cutaneous irAEs was 104.9 ± 168.1 days. The most frequent occurrence of cutaneous irAEs were maculopapular rash (29.9%), followed by eczematous conditions (20.9%) and erythema multiforme (11.9%). As the most severe irAEs, four cases (3.0%) of SJS/TEN were observed. None of the patients died from cutaneous irAEs. The mean BMI was 22.6 ± 4.0, and 19 patients had a BMI < 18.5, the criteria for underweight (14.1%), while CT imaging analysis revealed that 65 patients (50.4%) had sarcopenia at the initiation of ICI. Analysis according to severity grade showed that the interval between ICI initiation and the onset of cutaneous irAEs tended to be shorter in patients with grade 4 irAEs (49.1 ± 51.4 days) than in those with lower‐grade irAEs; however, the differences among grades were not statistically significant. Furthermore, SJS/TEN and erythema multiforme were more frequently observed in grade 4 cutaneous irAEs, whereas maculopapular rash, eczematous conditions, and psoriasiform eruptions were more commonly observed in grades 1–3. Grade 4 was characterized by a higher percentage of females (44.4%) and sarcopenia (72.2%) than the other grades. The most common grade 3 and 4 cutaneous irAEs that required systemic corticosteroid therapy, hospitalization, and discontinuation of ICIs were erythema multiforme and SJS/TEN (64.3%). No differences were observed based on the type of malignancy or ICIs. In the analysis of laboratory data, the WBC, neutrophil, and monocyte counts and the neutrophil‐to‐lymphocyte ratio tended to be higher in grade 4 than in the other grades.

**TABLE 5:** Comparison of clinical characteristics of patients with cutaneous irAEs by grade.

Variable	Total	Grade 1	Grade 2	Grade 3	Grade 4	*p*
Number of cutaneous irAEs, n	134	52	54	10	18	
Age at initiation of ICI administration (median), year (IQR)	66.0 (59.0–75.0)	70.0 (62.8–75.0)	63.5 (55.3–72.8)	55.5 (54.3–65.3)	67.5 (65.0–75.8)	0.015
Gender, *n* (%)						
Male	98 (76.0)	44 (84.6)	38 (70.4)	9 (90.0)	10 (55.6)	0.044
Female	31 (24.0)	8 (15.4)	16 (29.6)	1 (10.0)	8 (44.4)	
BMI (mean ± SD)	22.6 ± 4.0	22.8 ± 4.1	22.6 ± 4.0	22.5 ± 3.9	21.6 ± 3.2	0.727
BMI < 18.5, *n* (%)	19 (14.1)	7 (13.5)	6 (11.1)	3 (30)	3 (16.7)	0.460
Sarcopenia, *n* (%)	65 (50.4)	26 (50.0)	25 (46.3)	1 (10)	13 (72.2)	0.017
Interval between initiation of administration and the onset (days) (mean ± SD)	104.9 ± 168.1	130.4 ± 198.6	100.9 ± 165.6	94.1 ± 106.7	49.1 ± 51.4	0.361
Causative ICIs, *n* (%)						
Nivolumab	47 (31.7)	17 (32.7)	15 (27.8)	2 (20.0)	6 (33.3)	
Pembrolizumab	48 (32.4)	20 (38.5)	19 (35.2)	3 (30.0)	3 (16.7)	
Atezolizumab	7 (4.7)	4 (7.7)	0 (0)	0 (0)	1 (5.6)	
Durvalumab	8 (5.4)	5 (9.6)	2 (3.7)	0 (0)	0 (0)	
Avelumab	1 (0.7)	1 (1.9)	0 (0)	0 (0)	0 (0)	
Ipilimumab	1 (0.7)	0 (0)	0 (0)	0 (0)	0 (0)	
Nivolumab + Ipilimumab	36 (24.3)	5 (9.6)	18 (33.3)	5 (50.0)	8 (44.4)	
Type of cutaneous irAEs, *n* (%)						
Maculopapular rash	40 (29.9)	0 (0)	38 (70.4)	2 (20.0)	0 (0)	
Eczematous conditions	28 (20.9)	18 (34.6)	8 (14.8)	2 (20.0)	0 (0)	
Erythema multiforme	16 (11.9)	0 (0)	2 (3.7)	2 (20.0)	12 (66.7)	
Lichen planus	10 (7.5)	5 (9.6)	1 (1.9)	2 (20.0)	2 (11.1)	
Psoriasiform eruption	8 (6.0)	4 (7.7)	4 (7.4)	0 (0)	0 (0)	
SJS/TEN	4 (3.0)	0 (0)	0 (0)	0 (0)	4 (22.2)	
Bullous diseases	4 (3.0)	3 (5.8)	0 (0)	1 (10.0)	0 (0)	
Purpuric eruption	2 (1.5)	2 (3.8)	0 (0)	0 (0)	0 (0)	
Other	22 (16.4)	20 (38.5)	1 (1.9)	1 (10.0)	0 (0)	
Type of malignancies, *n* (%)						
Renal cancer	34 (25.4)	8 (15.4)	19 (35.2)	0 (0)	7 (38.9)	
Melanoma	24 (17.9)	8 (15.4)	8 (14.8)	5 (50.0)	3 (16.7)	
Lung cancer	16 (11.9)	5 (9.6)	7 (13.0)	2 (20.0)	2 (11.1)	
Pharyngeal cancer	12 (9.0)	9 (17.3)	2 (3.7)	0 (0)	1 (5.6)	
Gastric cancer	7 (5.2)	3 (5.8)	2 (3.7)	0 (0)	2 (11.1)	
Esophageal cancer	7 (5.2)	2 (3.8)	3 (5.6)	0 (0)	2 (11.1)	
Hepatocellular carcinoma	7 (5.2)	6 (11.5)	1 (1.9)	0 (0)	0 (0)	
Cervical cancer	6 (4.5)	0 (0)	5 (9.3)	1 (10.0)	0 (0)	
Bladder cancer	5 (3.7)	2 (3.8)	3 (5.6)	0 (0)	0 (0)	
Oral cancer	4 (3.0)	3 (5.8)	0 (0)	1 (10.0)	0 (0)	
Bile duct cancer	3 (2.2)	2 (3.8)	1 (1.9)	0 (0)	0 (0)	
Paranasal cancer	2 (1.5)	1 (1.9)	0 (0)	0 (0)	1 (5.6)	
Malignant lymphoma	2 (1.5)	0 (0)	2 (3.7)	0 (0)	0 (0)	
Colorectal cancer	1 (0.7)	0 (0)	0 (0)	1 (10.0)	0 (0)	
Neuroendocrine carcinoma	1 (0.7)	1 (1.9)	0 (0)	0 (0)	0 (0)	
Uterine cancer	1 (0.7)	1 (1.9)	0 (0)	0 (0)	0 (0)	
Neuroblastoma	1 (0.7)	0 (0)	1 (1.9)	0 (0)	0 (0)	
Pleural mesothelioma	1 (0.7)	1 (1.9)	0 (0)	0 (0)	0 (0)	
Laboratory data (mean ± SD)						
White blood cells (/μL)	6831 ± 3281	6153 ± 2434	7011 ± 3686	6240 ± 987	8577 ± 4133	0.049
Neutrophils (/μL)	4712 ± 3113	4073 ± 2230	4889 ± 3535	4369 ± 779	6217 ± 4046	0.083
Lymphocytes (/μL)	1319 ± 555	1294 ± 542	1334 ± 488	1171 ± 556	1427 ± 732	0.684
Monocytes (/μL)	551 ± 250	516 ± 222	547 ± 254	476 ± 90	703 ± 312	0.035
Eosinophils (/μL)	213 ± 234	221 ± 211	186 ± 140	182 ± 92	292 ± 455	0.399
Platelet (/μL)	282 000 ± 137 000	278 000 ± 155 000	285 000 ± 137 000	278 000 ± 56 500	285 000 ± 110 000	0.993
CRP (mg/dl)	2.08 ± 4.22	1.83 ± 4.75	1.67 ± 3.27	2.58 ± 3.83	3.78 ± 4.85	0.770
Albumin (g/dl)	3.8 ± 0.7	3.9 ± 0.6	3.7 ± 0.8	3.7 ± 0.4	3.6 ± 0.7	0.476
NLR	4.7 ± 5.5	3.8 ± 2.9	4.6 ± 4.5	4.6 ± 1.9	8.1 ± 11.0	0.043
LMR	2.8 ± 1.5	2.8 ± 1.2	2.9 ± 1.7	2.4 ± 0.9	2.4 ± 1.5	0.486
CAR	0.9 ± 2.4	0.7 ± 2.2	0.9 ± 2.7	0.8 ± 1.2	1.4 ± 2.3	0.770
PLR	2764 ± 2871	2706 ± 2539	2398 ± 1366	3035 ± 1490	3881 ± 5848	0.3000
Patients' treatment histories, *n* (%)						
Chemotherapy prior to ICIs initiation	34 (25.4)	13 (25.0)	14 (25.9)	4 (40.0)	3 (16.7)	0.601
Combination with chemotherapy at the initiation of ICIs treatment	37 (27.6)	14 (26.9)	15 (27.8)	3 (30.0)	5 (27.8)	0.998
Outcome, *n* (%)						
Dead	66 (51.2)	25 (48.1)	28 (51.9)	2 (20.0)	11 (61.1)	0.396
Survive	62 (48.1)	27 (51.9)	25 (46.3)	8 (80.0)	7 (38.9)	
Unknown	1 (0.8)	0 (0)	1 (1.8)	0 (0)	0 (0.0)	

Abbreviations: BMI, body mass index; CAR, CRP‐to‐albumin ratio; ICIs, immune checkpoint inhibitors; IQR, interquartile range; irAE, immune‐related adverse events; LMR, lymphocyte‐to‐monocyte ratio; NLR, neutrophil‐to‐lymphocyte ratio; PLR, platelet‐to‐lymphocyte ratio; SD, standard deviation; SJS, Stevens–Johnson syndrome; TEN, toxic epidermal necrolysis.

To further identify the risk factors for developing grade 4 cutaneous irAEs, we performed multivariable analysis. The results showed that female sex (OR = 4.13; 95% CI, 1.36–13.11; *p* = 0.012) and elevated baseline monocyte count (OR = 6.68; 95% CI, 1.87–27.61; *p* = 0.003) were significantly associated with the development of grade 4 cutaneous irAEs (Table 6). However, no statistical significance was noted for the ICI type.

**TABLE 6:** Multivariable logistic regression for the risk of the severity of cutaneous irAEs.

Variable	OR	95% CI	*p*
Female	4.13	1.36–13.11	0.012
Type of ICIs			
PD‐1 + CTLA‐4 vs. PD‐1	2.55	0.84–7.80	0.096
PD‐1 + CTLA‐4 vs. PD‐L1	1.84	0.32–19.41	0.517
PD‐1 vs. PD‐L1^a^	0.72	0.13–7.47	0.744
Laboratory data^b^			
Monocytes	6.68	1.87–27.61	0.003

Abbreviations: CI, confidence interval; CTLA‐4, Cytotoxic T‐lymphocyte Antigen‐4; ICIs, immune checkpoint inhibitors; irAE, immune‐related adverse events; OR, odds ratio; PD‐1, Programmed cell Death 1; PD‐L1, Programmed cell Death ligand 1.

^a^ PD‐1 vs. PD‐L1 was obtained from the same model.

^b^ Monocytes were log(1 + x) transformed.

## 4 Discussion

This study examined the clinical and biological data at the initiation of ICIs to identify the factors associated with the incidence and risk of cutaneous irAEs in the Japanese population. Statistical analyses revealed that anti‐PD‐1 and anti‐CTLA‐4 combination therapy, melanoma, and a high baseline eosinophil count were significantly associated with the risk of developing cutaneous irAEs. Furthermore, female sex and high baseline monocyte counts were identified as risk factors for severe cutaneous irAEs. To the best of our knowledge, this is the first retrospective Japanese study to investigate the association between risk factors and the development and severity of cutaneous irAEs.

Previous studies have identified several risk factors for cutaneous irAE. Pre‐existing inflammatory skin diseases, such as atopic dermatitis and psoriasis, are associated with an increased risk, and patients with these conditions tend to develop more severe cutaneous irAEs [19]. Furthermore, anti‐PD‐1 and anti‐CTLA‐4 combination therapy is associated with an increased risk of pruritus and skin rash compared with monotherapy, which is consistent with our findings [20]. A systematic review revealed that the frequency of cutaneous irAEs depends on the type of malignancy, with melanoma being associated with a higher risk of developing cutaneous irAEs than NSCLC or renal cancer [7]. A recent report indicated that melanoma, lung cancer, and renal cancer are associated with a higher risk of developing cutaneous irAEs, and, as clinical types, melanoma and urothelial carcinoma increase the incidence of vitiligo and SJS, respectively [21]. Moreover, our multivariable analysis demonstrated that melanoma was significantly associated with the development of cutaneous irAEs, consistent with previous reports. In contrast, no statistically significant associations were observed for lung or renal cancer. This difference may be attributable to racial variation, as previous studies analyzing international cohorts or combining data from multiple countries have shown that Asians are more susceptible to ICI‐related kidney injury than Caucasians [22, 23]. Furthermore, patients with metastatic cancer who received ICIs treatment were significantly associated with human leukocyte antigen (HLA)‐DRB1*11:01 and pruritus, suggesting a genetic etiology for irAEs [24]. HLA distribution differs between racial groups, and individual differences in HLA genes affect the immune response, which may have influenced the results.

Furthermore, we observed that elevated baseline peripheral blood eosinophil counts were associated with the development of cutaneous irAEs. The use of blood count biomarkers to predict irAE development has been previously reported. Michailidou et al. reported statistically significant differences in the development of irAEs in patients with an absolute lymphocyte count > 2600/μL, monocyte count > 290/μL, and platelet count > 145 000 /μL at baseline [25]. Although an analysis of the association between eosinophil counts and the development of irAEs was not analyzed in that study, these findings highlight the potential utility of blood count biomarkers in predicting irAEs.

To date, cutaneous irAEs are thought to occur via several immunological mechanisms. A representative mechanism involves PD‐1 blockade disrupting peripheral tolerance of CD8^+^ T cells to cutaneous neoantigens, leading to their activation and keratinocyte injury that drives cutaneous irAEs [26]. Other mechanisms, including B‐cell activation, autoantibody production, and inflammatory cytokine networks, may also contribute to the pathogenesis of cutaneous irAEs, depending on the clinical subtype [16]. Therefore, although some cutaneous irAEs may share certain features with delayed immune‐mediated drug reactions, a high peripheral eosinophil count should be regarded as only one potential factor associated with the development of cutaneous irAEs. In this study, a higher baseline eosinophil count was independently associated with the development of cutaneous irAEs, and eosinophil counts further increased at the onset of these events. These findings suggest that eosinophils may contribute to the immunological environment that predisposes patients to cutaneous irAEs. However, our analysis revealed no significant differences in the prevalence of allergic diseases between patients with and without cutaneous irAEs, suggesting that baseline eosinophilia may reflect a broader inflammatory or immune state rather than a specific allergic predisposition. Moreover, eosinophilia and eosinophil‐associated severe cutaneous adverse reactions have also been reported in patients receiving non‐ICI anticancer agents, indicating that eosinophil elevation is not specific to allergic reactions associated with ICIs [27].

In addition to the risk factors for the onset of cutaneous irAEs, female sex and high monocyte count at baseline were associated with grade 4 cutaneous irAEs. These findings may reflect a pro‐inflammatory immune background that predisposes patients to severe cutaneous inflammation after ICI treatment. Sex‐based differences in immune responses are well recognized, with females generally exhibiting stronger innate and adaptive immune responses than males [28, 29]. These differences may be influenced by sex hormones, X chromosome‐linked immune regulatory genes, and epigenetic regulation [29]. Sex hormones can also modulate monocyte/macrophage function, including antigen presentation and the production of inflammatory cytokines and chemokines [30]. Therefore, sex‐related differences in immune regulation may contribute to enhanced monocyte/macrophage‐driven inflammatory responses following ICI treatment. Monocytes and macrophages may also contribute directly to severe cutaneous irAEs. Activated monocytes/macrophages can produce inflammatory mediators such as IL‐1β, IL‐6, TNF‐α, IL‐15, and CXCL9/10, which promote T‐cell recruitment and cytotoxic immune responses. Previous studies have shown that high monocyte counts are associated with irAE development [25] and that CD14^+^ and CD16^+^ monocyte populations increase in patients with severe irAEs [31]. These findings suggest that a high baseline monocyte count may reflect a pre‐existing inflammatory myeloid state that is further amplified by ICI treatment. This pathway may be particularly relevant to grade 4 cutaneous irAEs, including SJS/TEN. Recent studies have shown that ICI‐induced severe epidermal necrolysis may be mediated by macrophage‐derived CXCL10, which recruits CXCR3^+^ cytotoxic T lymphocytes to affected skin, while TNF signaling further contributes to this inflammatory cascade [32]. In addition, studies of PD‐1 inhibitor‐induced SJS/TEN have demonstrated significantly elevated TNF‐α levels in affected skin, with macrophages identified as the predominant immune cell population and the primary source of TNF‐α within blister lesions [33]. Therefore, a high baseline monocyte count may indicate a pre‐existing myeloid inflammatory state that promotes excessive macrophage activation and TNF‐α/CXCL10‐mediated recruitment of cytotoxic lymphocytes, ultimately resulting in severe keratinocyte injury following ICI administration. However, this proposed mechanism remains speculative because cytokine profiling, flow cytometric analyses, and lesional tissue studies were not performed in the present study.

Furthermore, a recent meta‐analysis showed that the presence of sarcopenia before immunotherapy in patients with malignancy was associated with poor clinical outcomes [34]. However, an increased incidence and severity of irAEs have not been observed in patients with malignancies and sarcopenia [34, 35]. To date, no studies have examined the relationship between sarcopenia and cutaneous irAEs. We analyzed the association between sarcopenia and the severity of cutaneous irAEs but found no significant relationship. Therefore, our results suggest that pre‐existing sarcopenia may not influence the severity of cutaneous irAEs.

Our study has some limitations. First, this was a single‐center retrospective study. Therefore, there may be a bias in the type of malignancy treated and the choice of ICIs. Second, the incidence of grades 1 and 2 cutaneous irAEs may be underestimated because it is possible that some patients were treated in the clinic rather than in our hospital due to the mild severity of the cutaneous symptoms. This may have led to a lower incidence of cutaneous irAEs in this study than in previous studies [36, 37]. Third, we demonstrated that baseline eosinophil and monocyte counts were associated with the development and severity of cutaneous irAEs, independent of prior or concurrent chemotherapy. However, we were unable to analyze detailed treatment‐related factors, such as specific chemotherapy regimens or combination strategies with ICIs. Therefore, the potential influence of these factors cannot be completely excluded. Finally, this study defined cutaneous irAEs as cutaneous manifestations that occurred during ICIs administration and were clinically diagnosed by dermatologists. Therefore, ICIs have not been identified as the causative agents. In addition, patients receiving ICIs used various medications simultaneously, which may have contributed to the onset of cutaneous irAEs. Furthermore, in recent cancer therapies, ICIs have been administered in combination with multiple cytotoxic chemotherapeutic agents. Therefore, this study may have included patients in whom ICIs were not the cause of cutaneous manifestations.

This study has several strengths. Our population consisted only of Japanese individuals, which could have eliminated the racial factors. In addition, all cutaneous irAEs were diagnosed and treated by a dermatologist, allowing for the collection of detailed data, including blood test results, treatment details, and outcomes. Furthermore, several of our findings align with previously reported data, suggesting that, with further validation, blood cell counts and sex may serve as useful biomarkers for predicting the development and severity of cutaneous irAEs.

In conclusion, our findings demonstrate that peripheral blood test data, including eosinophil and monocyte counts at the initiation of ICI, female sex, melanoma, and anti‐PD‐1 and anti‐CTLA‐4 combination therapy, may be potential risk factors for the development and severity of cutaneous irAEs in the Japanese population. Further validation of our findings may contribute to a more accurate estimation of cutaneous irAEs and improve the prognosis of patients with malignancies.

## Funding

The authors have nothing to report.

## Ethics Statement

Approval of the Research Protocol by an Institutional Review Board: The study protocol was approved by the institutional review board of Yokohama City University (approval no.: F241100033).

## Consent

This study was conducted using an opt‐out approach in accordance with the Ethical Guidelines for Medical and Health Research Involving Human Subjects in Japan. Information about the study was disclosed to all potential participants, and opportunities to decline participation were provided.

## Conflicts of Interest

The authors declare no conflicts of interest.

## Supporting information


**Table S1:** the clinical subtypes of cutaneous irAEs in 11 patients who developed multiple cutaneous irAEs.
**Table S2:** Analysis of changes in eosinophil count during the initiation of ICIs administration and the onset of cutaneous irAEs in 145 cases, categorized by clinical subtype.
**Figure S1:** Comparison of baseline eosinophil counts at baseline in each cutaneous irAEs.
EC, eczematous conditions; EM, erythema multiforme; LP, lichen planus; MP, maculopapular rash; Pso, psoriasiform eruption; SJS, Stevens‐Johnson syndrome; TEN, toxic epidermal necrolysis.

## Data Availability

The data that support the findings of this study are available from the corresponding author upon reasonable request.
