# Supplementary material for: Risk Factors for Cutaneous Immune‐Related Adverse Events in the Japanese Population: A Retrospective Single‐Center Cohort Study
Source: J Dermatol. 2026 Jul 2;53(9):1245–60. doi: 10.1111/1346-8138.70370 (PMC13555148; doi:10.1111/1346-8138.70370)
Supplement: Supplementary file 1 [file JDE-53-1245-s001.docx]

Table S1. The clinical subtypes of cutaneous irAEs in eleven patients who developed multiple cutaneous irAEs.

| The combinations of cutaneous irAEs | Number of patients |
| --- | --- |
| Double cutaneous irAEs |  |
| Eczematous conditions and vitiligo | 3 |
| Maculopapular rash and vitiligo | 2 |
| Erythema multiforme and maculopapular rash | 1 |
| Erythema multiforme and eczematous conditions, | 1 |
| Erythema multiforme and lichen planus | 1 |
| Lichen planus and vitiligo, | 1 |
| Maculopapular rash and other | 1 |
| Triple cutaneous irAEs |  |
| Maculopapular rash, lichen planus, and vitiligo | 1 |

irAE: immune-related adverse events

Table S2. Analysis of changes in eosinophil count during the initiation of ICIs administration and the onset of cutaneous irAEs in 145 cases, categorized by clinical subtype.

| The clinical subtype of  cutaneous irAEs | Number of  cases | Eosinophil count  at baseline (/μL) | Eosinophil count  at onset (/μL) |
| --- | --- | --- | --- |
| Maculopapular rash | 40 | 196 ± 145 | 392 ± 473 |
| Eczematous conditions | 28 | 199 ± 168 | 431 ± 624 |
| Erythema multiforme | 16 | 169 ± 119 | 326 ± 323 |
| Lichen planus | 10 | 192 ± 250 | 593 ± 730 |
| Psoriasiform eruption | 8 | 169 ± 114 | 286 ± 269 |
| SJS/TEN | 4 | 753 ± 775 | 228 ± 278 |
| Bullous diseases | 4 | 411 ± 315 | 434 ± 264 |
| Purpuric eruption | 2 | 80 ± 3 | 188 ± 68 |
| Vitiligo | 11 | 165 ± 93 | 366 ± 254 |
| Other | 22 | 202 ± 156 | 324 ± 235 |

irAE: immune-related adverse events, SJS: Stevens-Johnson syndrome,

TEN: toxic epidermal necrolysis

Fig. S1 Comparison of baseline eosinophil counts at baseline in each cutaneous irAEs.

MP: maculopapular rash, EM: erythema multiforme, EC: eczematous conditions, SJS: Stevens-Johnson syndrome, TEN: toxic epidermal necrolysis, Pso: psoriasiform eruption, LP: lichen planus
